# Enteral nutrition therapy for elderly patients with common-type COVID-19, a retrospective study based on medical records

**DOI:** 10.1093/inthealth/ihaf012

**Published:** 2025-03-31

**Authors:** Jianming Yuan, Yuening Guan, Zhifeng Zhao, Jiankang Shen, Dan Tan, Fang Zhao, Lei Ge, Rongli Xie, Tingting Li

**Affiliations:** Department of General Surgery, Ruijin Hospital Luwan Branch, Shanghai Jiaotong University School of Medicine, 149 Chongqing South Road, Huangpu District, Shanghai, China; Department of General Surgery, Ruijin Hospital Luwan Branch, Shanghai Jiaotong University School of Medicine, 149 Chongqing South Road, Huangpu District, Shanghai, China; Department of Gastrointestinal Surgery, Xijing Hospital, Fourth Military Medical University, No.127, West Changle Road, Xi’an, Shaanxi Province, China; Department of General Surgery, Ruijin Hospital Luwan Branch, Shanghai Jiaotong University School of Medicine, 149 Chongqing South Road, Huangpu District, Shanghai, China; Department of General Surgery, Ruijin Hospital Luwan Branch, Shanghai Jiaotong University School of Medicine, 149 Chongqing South Road, Huangpu District, Shanghai, China; Department of General Surgery, Ruijin Hospital Luwan Branch, Shanghai Jiaotong University School of Medicine, 149 Chongqing South Road, Huangpu District, Shanghai, China; Department of General Surgery, Ruijin Hospital Luwan Branch, Shanghai Jiaotong University School of Medicine, 149 Chongqing South Road, Huangpu District, Shanghai, China; Department of General Surgery, Ruijin Hospital Luwan Branch, Shanghai Jiaotong University School of Medicine, 149 Chongqing South Road, Huangpu District, Shanghai, China; Department of General Surgery, Ruijin Hospital Luwan Branch, Shanghai Jiaotong University School of Medicine, 149 Chongqing South Road, Huangpu District, Shanghai, China

**Keywords:** COVID-19, elderly patients, enteral nutrition

## Abstract

**Background:**

The objective was to investigate the implications of enteral nutrition for elderly patients with common-type coronavirus disease 2019 (COVID-19).

**Methods:**

Data were retrospectively extracted from medical records. Enteral nutritional supplementation was recommended for patients with a nutritional risk score >3. The preferred method was oral administration, and preparations included Ensure and TPF-T. Continuous variables were compared using analysis of two-tailed Student's t-tests or one-way analysis of variance for normally distributed data and the rank sum test for non-normally distributed data. Categorical variables were compared using the χ^2^ test or Fisher’s exact test. Values of p <0.05 were considered to be statistically significant.

**Results:**

The mortality rate in the whole cohort was 9.54%. A total of 474 patients tested negative and were discharged; among them, 173 patients received enteral nutrition while 301 patients did not. There were significant correlations between mortality and age, serum albumin concentration, prognostic nutritional index, underlying severe disease status and diet condition. In patients with a poor diet, early use of enteral nutrition is associated with faster conversion to a negative polymerase chain reaction test.

**Conclusions:**

The prognosis of elderly patients with common-type COVID-19 was related to their nutritional status. Enteral nutritional supplementation is the preferred method of nutrition because it is the simplest and most widely accepted method for patients. For patients with poor diet conditions, enteral nutritional intervention should be performed early.

In December 2019, coronavirus disease 2019 (COVID-19) emerged suddenly and spread rapidly, causing a global pandemic.^1,2^ The virus continues to mutate, creating an enormous challenge for the world due to its continued outbreaks. Currently, the main prevalent strain worldwide is the Omicron variant.^3^ The Omicron strain, although less virulent than the Delta strain, is more prevalent than the other strains and has a high rate of mortality in elderly patients.

In designated hospitals for COVID-19 pneumonia, elderly patients account for a large proportion of patients. Among all pneumonia patients, the elderly individuals often have underlying medical conditions (including diabetes, hypertension, cardiovascular disease, chronic obstructive pulmonary disease etc.) and a very high mortality rate. The reasons for this occurrence are clinically controversial. Nutritional therapy is one of the key methods used to treat COVID-19 pneumonia patients.^4^ Improvement of nutrition intake of COVID-19 pneumonia patients includes nutrition education, oral nutrition supplementation, enteral nutrition (EN) supplementation and parenteral nutrition. For elderly patients, nutrition education has little effect and requires great human resources. In view of poor cardiopulmonary function, parenteral nutrition has a great burden on cardiac function, which is not preferred. EN supplementation is the easiest and most acceptable nutritional method for patients.^5–7^ The aim of this study was to review the nutrition-related indicators and the application of EN in elderly patients >75 y of age with common-type COVID-19 and to investigate the positive significance of EN in the treatment of elderly patients with common-type COVID-19.

## Methods

### Clinical information

A total of 524 elderly (enrolment age was ≥75 y) patients were included, with an average age of 86.93±6.54 y. All patients were diagnosed with common-type COVID-19 in our hospital between April 2022 and June 2022. Common-type COVID-19 was defined on the basis of clinical manifestations and imaging-confirmed pneumonia, according to the guidelines of the COVID-19 pneumonia treatment protocol (9th edition) issued by the National Health Commission.^4^

The nutritional risk score (NRS) and prognostic nutritional index (PNI) were used to assess nutritional risk and prognosis. EN supplementation was recommended for patients with an NRS >3. With the aim of identifying patients at risk, the NRS was developed as a prospectively validated screening tool. Nutritional status was evaluated by three variables: body mass index, recent weight loss and food intake during the last week before evaluation. An NRS >3 was considered to indicate the need for EN supplementation.^8,9^ In addition, the PNI is a novel inflammatory marker that is useful for predicting the prognosis of patients with certain conditions and is calculated as follows: PNI=10×serum albumin level (g/100 ml)+0.005×total lymphocyte count/mm^3^.^10^

The preferred method was oral administration, with preparations including Ensure (4.5 kcal/g; Abbott Laboratories, Abbott Park, IL, USA) and TPF-T (1.3 kcal/ml; Sino-Swed Pharmaceutical, Beijing, China). Patients with swallowing disorders and other conditions making it difficult to meet their energy requirements were fed by nasal feeding, with preparations such as TPF-T and TPF (1 kcal/ml; Nutricia, Amsterdam, The Netherlands).

### Data collection

Data were retrospectively extracted from medical records of Ruijin Hospital Lu Wan Branch. The hospital information system was used to retrospectively collect clinical data, including medical history, nursing records, patient age, sex, diet condition (within 3 d of admission), EN implementation situation and laboratory results such as the lymphocyte count, albumin concentration and the time point at which nucleic acid test results became negative.

The time point at which patients’ polymerase chain reaction (PCR) test results became negative was defined as two consecutive negative nucleic acid tests or a cycle threshold value ≥35 at an interval of >24 h.

This retrospective study was approved by the Ethics Committee of Ruijin Hospital Lu Wan Branch, Shanghai Jiaotong University School of Medicine. All the procedures were implemented based on the principles of the Declaration of Helsinki. Desensitization data were retrospectively extracted from the medical records. Since the data were retrospective and anonymized, patient consent was waived by the Ethics Committee of Ruijin Hospital Lu Wan Branch, Shanghai Jiaotong University School of Medicine.

### Statistical analysis

The statistical analysis was performed using SPSS version 21.0 (IBM, Armonk, NY, USA). Continuous variables are expressed as mean±standard deviation (SD) for normally distributed data. Continuous variables were compared using analysis of two-tailed Student's t-tests or one-way analysis of variance for normally distributed data and the rank sum test for non-normally distributed data. Categorical variables were compared using the χ^2^ test or Fisher’s exact test. Tests for the assumptions of normality distribution and variance homogeneity were then performed. The multivariate regression model was followed by a collinearity analysis between variables. Values of p <0.05 were considered to be statistically significant.

## Results

### Analysis of factors associated with mortality

As shown in Table 1, in the whole group of common-type COVID-19 patients, 474 patients tested negative and were discharged; 50 patients had a fatal prognosis, for a mortality rate of 9.54%. Age was significantly greater in the mortality group than in the rehabilitation group, while no significant difference was found in sex or the haemoglobin concentration between the two groups. The mortality group had a greater number of patients with severe underlying disease that was not severe pneumonia, which lead to poor nutritional status and thus a lower prognosis in pneumonia. For nutrition-related indicators, the peripheral blood leucocyte count and neutrophil count were significantly greater in the mortality group than in the rehabilitation group, and the peripheral blood lymphocyte count, albumin concentration and PNI were significantly lower. Patient mortality was significantly associated with the diet condition, and all patients in the mortality group ate <1/3 of their daily amount at the time of admission.

**Table 1.** Analysis of factors related to patients’ prognosis

Items	Lethal outcome group	Rehabilitation group	p-Value	χ^2^/t
Patients	50	474		
Sex, *n* (%)				
Male	22 (4.20)	175 (33.40)	0.358	0.996
Female	28 (5.34)	299 (57.06)		
Age (years), mean±SD	88.52±5.74	86.76±6.60	0.047	−2.026
WBC count (10^9^/l), mean±SD	9.83±5.04	6.49±3.18	0.000	−5.765
Neutrophils (10^9^/l), mean±SD	7.83±4.78	4.52±2.98	0.000	−6.095
Lymphocytes (10^9^/l), mean±SD	0.89±0.57	1.34±0.61	0.000	4.27
Haemoglobin (g/l), mean±SD	115.11±22.35	116.84±21.50	0.643	0.463
Albumin (g/l), mean±SD	29.47±10.40	34.87±9.63	0.001	3.202
PNI, mean±SD	33.80±12.05	41.16±11.34	0.000	3.670
Underlying diseases, n (%)				
Mild	2 (0.38)	349 (66.60)	0.000	98.963
Serious	48 (9.16)	125 (23.85)		
Diet condition, n (%)				
<1/3	50 (9.54)	155 (29.58)	0.000	66.060
1/3–2/3	0 (0.00)	174 (33.21)		
≥2/3	0 (0.00)	145 (27.67)		

White blood cell: WBC.

Diet condition <1/3 means the enrolled patients ate <1/3 of their daily amount since admission. Diet condition ≥2/3 means the enrolled patients ate >2/3 of their daily amount since admission.

### Analysis of factors associated with the rate of PCR negativity in patients

The correlations between the general clinical data, laboratory results, diet condition and EN implementation in convalescent elderly common-type COVID-19 patients and the rate of PCR negativity are shown in Table 2. There were no significant differences in sex, age, peripheral blood leucocyte count, neutrophil count or rate of PCR negativity. When grouped by lymphocyte count, 152 patients had a lymphocyte count <1.0×10^9^/l and 308 patients had a lymphocyte count equal to or greater than the aforementioned value. The number of days to turn negative was significantly greater in the low lymphocyte group than in the high lymphocyte group. Similar results were found for the serum albumin concentration and PNI. Furthermore, many patients had poor dietary conditions: 155 patients ate <1/3 of the daily amount, 174 patients ate between 1/3 and 2/3 of the daily amount and only 145 patients ate >2/3 of the daily amount within 3 d of admission. The multivariate regression model showed that the diet condition was significantly correlated with virus elimination (Tables 3 and 4). The rate of PCR negativity was significantly greater in patients who ate well than in those who ate poorly. According to the previously mentioned guidelines, EN supplementation was recommended for patients with an NRS >3. The results showed that for patients with poor diet, early use of EN supplementation was associated with faster conversion to a negative PCR test.

**Table 2.** Analysis of factors associated with the rate of PCR negativity in patients

Items		n	Change to negative results (since admission, days), mean±SD	p-Value	t/F
Sex	Male	175	11.47 ± 7.61	0.316	−1.004
	Female	299	10.79 ± 6.84		
Age (years)	<85	154	10.44 ± 7.30	0.207	1.263
	≥85	320	11.33 ± 7.04		
WBC count (10^9^/l)	<4	77	12.53 ± 7.39	0.169	1.791
	4–10	334	11.02 ± 7.18		
	>10	49	10.35 ± 6.60		
Neutrophils (10^9^/l)	<1.5	22	12.82 ± 6.93	0.551	0.597
	1.5–8	396	11.13 ± 7.23		
	>8	42	10.98 ± 6.70		
Lymphocytes (10^9^/l)	<1.0	152	12.47 ± 6.99	0.007	−2.696
	≥1.0	308	10.57 ± 7.18		
Haemoglobin (g/l)	<110	146	11.44 ± 7.67	0.617	−0.500
	≥110	314	11.08 ± 6.93		
Albumin (g/l)	<40	260	12.95 ± 7.67	0.001	−3.498
	≥40	136	10.30 ± 6.01		
PNI	<40	110	13.59 ± 7.88	0.010	−2.6
	≥40	282	11.49 ± 6.92		
Diet condition	<1/3	155	14.27 ± 8.39	0.000	66.713
	1/3–2/3	174	12.24 ± 6.23		
	≥2/3	145	6.17 ± 2.90		
EN	Yes	173	12.65 ± 8.35	0.000	3.775
	No	301	10.12 ± 6.15		
EN start date	≤3 d	123	10.71 ± 7.84	0.000	5.124
	>3 d	50	17.42 ± 7.71		

White blood cell: WBC.

Diet condition <1/3 means the enrolled patients ate <1/3 of their daily amount since admission. Diet condition ≥2/3 means the enrolled patients ate >2/3 of their daily amount since admission.

**Table 3.** The multivariate regression model with the rate of PCR negativity

Characteristic	β	95% CI	p-Value
Diet condition	−4.7	−5.8 to −3.7	<0.001
EN	−0.94	−2.6 to 0.69	0.3
ALB			
Low	–	–	
High	−1.7	−3.3 to −0.13	0.034
LY			
Low	–	–	
High	−0.86	−2.3 to 0.57	0.2
WBC count			
Low	–	–	
medium	−1.7	−3.5 to 0.05	0.057
High	−5.2	−7.8 to −2.6	<0.001
PNI			
Low	–	–	
High	1.6	−0.07 to 3.2	0.060

CI: confidence interval; WBC: white blood cell.

**Table 4.** Collinear regression table

GVIF	df	GVIF(1/(2×df))	row.names
1.655311	1	1.286589	Diet condition
1.546574	1	1.243613	EN
1.28797	1	1.134888	ALB
1.137732	1	1.066645	LY
1.143795	2	1.034158	WBC
1.496576	1	1.223346	PNI

df: degrees of freedom; GVIF: generalized variance inflation factor.

### Stratified analysis by the diet condition of patients

A total of 155 patients ate <1/3 of their daily routine amount, of whom 114 received EN supplementation and 41 did not. Moreover, there was no significant difference between the recovery time in the EN group that received EN supplementation and that in the group that did not receive EN supplementation, while early EN supplementation significantly shortened the recovery time. A total of 174 patients consumed 1/3–2/3 of their daily food intake; 54 of these patients received EN supplementation and 120 did not receive EN supplementation and the time to negativity was shorter in the group that received EN than in the group that did not. Additionally, early EN supplementation significantly shortened the recovery time. A total of 145 patients ate >2/3 of their daily amount; 5 of these patients received EN supplementation, all of whom had EN supplementation implemented within 3 d of admission, and 140 did not receive EN supplementation. There was no significant difference in the rate of PCR negativity between the group that received EN supplementation and the group that did not receive EN supplementation. The results are shown in Table 5.

**Table 5.** Stratified analysis by the diet condition of patients

Items	n	The rate of PCR negativity (days), mean±SD	p-Value	t	
<1/3 of routine					
EN	Yes	114	14.36 ± 9.07	0.827	−0.219
	No	41	14.02 ± 6.18		
Early EN	Yes	81	12.10 ± 8.61	0.001	3.495
	No	74	16.65 ± 7.49		
1/3–2/3 of routine					
EN	Yes	54	9.70 ± 5.56	0.000	3.729
	No	120	13.38 ± 6.20		
Early EN	Yes	37	8.37 ± 5.43	0.000	4.472
	No	137	13.28 ± 6.03		
≥2/3 of routine					
EN	Yes	5	5.60 ± 2.19	0.659	0.442
	No	140	6.19 ± 2.93		

<1/3 means the enrolled patients ate <1/3 of their daily amount since admission. ≥2/3 means the enrolled patients ate ≥2/3 of their daily amount since admission.

### Stratified analysis by the PNI

The results of stratified analysis according to the PNI within 3 d after admission are shown in Table 6. There were 122 patients with a PNI <40; 72 patients received EN supplementation and 50 did not. Moreover, there was no significant difference in the rate of PCR negativity. There were 270 patients with a PNI ≥40, 71 of whom received EN and 199 of whom did not. The rate of PCR negativity was lower in the group that received EN than in the group that did not. However, recovery was not accelerated in the EN group with or without early implementation.

**Table 6.** Stratified analysis by PNI

Items	n	Rate of PCR change to negative (days), mean±SD	p-Value	t	
PNI <40					
EN	Yes	72	16.10 ± 8.42	0.226	1.218
	No	50	14.38 ± 6.41		
Early EN	Yes	41	14.12 ± 9.37	0.194	−1.306
	No	81	16.04 ± 6.63		
PNI ≥40					
EN	Yes	71	15.62 ± 8.68	0.003	3.048
	No	199	12.72 ± 6.13		
Early EN	Yes	46	12.65 ± 7.70	0.378	−0.883
	No	224	13.65 ± 6.84		

## Discussion

The Omicron variant is the most transmissible variant to date; although it is associated with a lower severe disease rate and mortality rate than the Delta variant,^11,12^ it still carries a greater risk in older patients.^13–15^ In our research, the mortality rate was as high as 9.54% among common-type COVID-19 elderly patients, which may be related to their old age and the fact that most of these patients had more severe underlying diseases.

Our study included a total of 524 elderly patients; 474 patients were discharged and 50 patients had a fatal prognosis, for a mortality rate of 9.54%. Analysis of the factors associated with lethal outcomes revealed significant correlations with age; peripheral blood leucocyte, neutrophil and lymphocyte counts; albumin concentration and underlying disease and diet status at admission. It is suggested that co-infection with bacteria, immune deficiency, severe underlying disease and malnutrition are high-risk factors for lethal outcomes in elderly patients with common-type COVID-19.

We considered the rate of PCR negativity as the main observation index, but it was found that two of the commonly used clinical indicators, peripheral blood lymphocyte count and albumin concentration, were significantly correlated with the main observation indices. Lymphocytes are a type of leucocyte that functions as a cell lineage with specific immune recognition functions in the body and play a pivotal role in immune processes through anti-infection and antitumor effects.^16,17^ The peripheral blood lymphocyte count can reflect the immune status of the body and is an important indicator of immune status. Additionally, albumin is the predominant protein within human plasma and reflects the nutritional status of the body. Several studies have suggested that the serum albumin concentration is negatively correlated with the NRS score,^18–20^ and Global Leadership Initiative on Malnutrition criteria have also been used in combination with other factors for the assessment of nutritional risk and determination of the severity of malnutrition.^21^ Studies have demonstrated that nutritional and immune status are closely related to the clinical outcomes of patients with various diseases; for instance, the PNI is commonly used to predict the risk of surgery and the prognosis of oncology patients.^22,23^ Moreover, Haines et al.^6^ demonstrated that the PNI is a good prognostic predictor for severe-type COVID-19 disease, and this study also demonstrated that the PNI is a predictor of the rate of PCR negativity in patients with common-type COVID-19.^24–26^

The results showed that the rate of PCR negativity was lower in patients in the control group. The same phenomenon was observed in the analysis stratified by PNI score, in which no benefit was observed for patients who received EN supplementation. However, in a stratified analysis of patients, it was discovered that EN supplementation significantly accelerated the rate of the PCR turning negative for those with a poor diet condition. In addition, early EN support after patient admission was superior to EN supplementation provided at a later stage, with more significant clinical benefits. Early proactive EN interventions should be provided for patients with common-type COVID-19 if they have poor diet conditions. In particular, the diet condition is considered a more valuable indication of EN support than the PNI.

## Conclusions

The prognosis of elderly patients with common-type COVID-19 was related to their nutritional status. EN supplementation is the preferred method of nutrition because it is the simplest and most widely accepted method for patients. For patients with poor diet conditions, EN intervention should be performed early.

Our patients with COVID-19 pneumonia were of older age and had more underlying medical conditions, which provides a unique advantage for this study. Moreover, given the unique nature of COVID-19 pneumonia affecting the whole body, it provides a critical therapeutic experience for other systemic diseases.

## Limitations

This study has several limitations. First, because the patients were all Chinese, there may be potential bias. Second, clinicians tend to administer EN support to more critically ill patients according to the severity of disease, diet condition and NRS,^27^ therefore there was an inevitable selection bias in this retrospective study. Additional data and a more reasonable stratified design, as well as multicentre randomized controlled trials, are needed to further validate our findings.

## Data Availability

The raw data supporting the conclusions of this article will be made available by the authors. Please contact the corresponding authors for additional data information.
